# Catalytic Degradation of Triphenylmethane Dyes with an Iron Porphyrin Complex as a Cytochrome P450 Model

**DOI:** 10.3390/molecules28145401

**Published:** 2023-07-14

**Authors:** Xiaoyan Lu, Qiman Che, Xinkai Niu, Yilin Zhang, Yu’e Chen, Qing Han, Miaoqing Li, Shuang Wang, Jihong Lan

**Affiliations:** 1 Henan Key Laboratory of Function-Oriented Porous Materials, College of Chemistry and Chemical Engineering, Luoyang Normal University, Luoyang 471934, China; 2 Xinjiang Production & Construction Corps Key Laboratory of Advanced Energy Storage Materials and Technology, College of Science, Shihezi University, Shihezi 832003, China; 3 School of Chemistry and Materials Engineering, Xinxiang University, Xinxiang 453003, China

**Keywords:** iron porphyrin complex, dye degradation, rhodamine B, malachite green, iodosobenzene, UV-vis

## Abstract

The organic dyes used in printing and dyeing wastewater have complex components, diverse structures and strong chemical stability, which make them not suitable for treatment and difficult to degrade in the environment. Porphyrins are macromolecules with 18 π electrons formed by four pyrrole molecules connected with a methylene bridge that has a stable structure. Porphyrin combines with iron to form an active intermediate with a structure similar to the cytochrome P450 enzyme, so they are widely used in the biomimetic field. In the current study, 5,10,15,20-tetra (4-carboxyphenyl) porphine ferric chloride (III) (Fe(III)TCPP) was used as a catalyst and iodosobenzene was used as an oxidant to explore the catalytic degradation of triphenylmethane dyes, such as rhodamine B (RhB) and malachite green (MG). The results of UV-Vis spectral analysis have shown that the conversion rate of the rhodamine B was over 90% when the amount of Fe(III)TCPP was 0.027 mM and the amount of iodosobenzene was eight equivalents. When the catalyst was 0.00681 mM and the amount of the oxidant was five equivalents, the conversion rate of the malachite green reached over 95%. This work provides a feasible method for the degradation of triphenylmethane dyes.

## 1. Introduction

Organic wastewater contamination has posed a serious risk of environmental hazards and toxicity to human health with the growth of the printing and dyeing industries, in which dyes were a medieval organic source of pollution [1]. Due to the increasing complexity of their components, if not handled properly, they will cause serious pollution and damage to the aquatic environment, thus threatening public health and the environment at large. Thus, the proper treatment of dye-containing wastewater is one of the biggest issues in water pollution prevention and the only way to develop sustainably [2]. Wastewater from printing and dyeing contains a variety of organic dyes, such as triphenylmethane dye, of which rhodamine B (RhB) and malachite green (MG) are representative examples. RhB is a synthetic organic dye with a complex structure and strong fluorescence and has been widely used in industrial dyeing, paper making, cosmetics and other industries. RhB is also an important representative of xanthene dyes and usually used as a dye laser material due to its good stability [3]. It is an allergen to the skin, the eyes and the respiratory system, and it is also a very well-recognized fluorescent water tracer [4]. MG is a crystalline solid and dark green, mainly used as an agent in the dyeing industry for the coloring of cotton fabric, paper and leather [5,6]. Both RhB and MG are very toxic and harmful to the environment. If discharged directly into the environment from industrial applications, they will affect the growth of plants and animals, resulting in serious water pollution and making the water resource problem more severe [7].

At present, various methods have been carried out to treat the dyeing waste from industrial wastewater. For example, activated carbon has been used for the absorption of water-soluble dyes, but since the activated carbon can easily reach saturation after absorption, it must be regenerated before it can be used again. Thus, this method is used for low-depth treatment or the treatment of a small amount of wastewater. Membrane separation technology uses a variety of different pore sizes of membranes, resulting in chemical products with different particle sizes being filtered out. The separation film can be used repeatedly, but the membranes are expensive to produce. Moreover, this physical disposal method is affected by the environment and cannot completely decompose or remove waste [8,9,10]. Photocatalytic oxidation is considered an emerging technology used to remove organic pollutants [3,11,12]. For example, crosslinked gelatin/CuS/PVA nanocomposites have proven to be effective and convenient photocatalysts for removing RhB dye from wastewater under solar irradiation [3]. Allé found that the photocatalytic degradation efficiency of an RhB synthetic solution was about 90%, having used TiO_2_ immobilized on SiC foam [13]. Reduced graphene-oxide-based, iron-oxide-modified titanium (rGO-Fe_3_O_4_/TiO_2_) has exhibited commendable photocatalytic efficiency (99%) comparatively to pure TiO_2_ (67%) under visible light for 55 min for MG degradation [14]. However, this method was limited, which resulted from the small active area of the photocatalysts, insufficient light absorption and instantaneous electron hole separation. Recent scientific studies have shown that peroxidases formed by some bacteria, such as laccases, lignin peroxidase (LiP) and manganese peroxidase (MnP), can be used as peroxide oxidation catalysts to achieve the effective decolorization of dyes at room temperature [10]. However, because natural enzymes cannot be extracted, their application in industrial production is also limited. Porphyrins consist of four pyrrole methylene α-carbon atoms connected to each other by a methylene bridge (=CH-) [15]. There are two pyrrole protons (N-H) in the porphyrin ring. When the two N-H atoms are replaced with different metal ions, various types of metal porphyrins will be generated. For example, the heme containing cytochrome is made up of complexes of iron porphyrins, vitamin B12 is made up of complexes of cobalt porphyrins and both of them have important physiological functions in biological bodies. Hemocyanin (cupric porphyrin) in animals, which exists in cytochromes, and chlorophyll (magnesium porphyrin) in plants play important roles in the oxygen-carrying respiration of blood cells and the photosynthesis of plant cells, respectively [16,17,18,19]. Thus, the research of simulated enzymes has aroused great interest and been developing rapidly [20,21,22,23,24,25,26]. An iron porphyrin complex is the general term for a complex formed of porphyrin and iron ions, exhibiting strong thermal stability and high chemical stability, that has broad application prospects in biomimetic catalysis, energy storage, probe detection and other fields. As the model of the P450 enzyme, an iron porphyrin complex can catalyze oxidation reactions in mild conditions, showing high catalytic efficiency and selectivity; thus, it has been of wide concern for scientific researchers [27].

A number of metal porphyrins have been used to catalyze the degradation of dyes [28,29,30,31,32,33,34,35,36]. Using *meso*-tetrakis(1-methylpyridinium-4-yl)porphyrinatomanganese(III) [Mn^III^(tmpyp)] as the catalyst, Meyer et al. investigated the degradation of dyes in an aqueous solution, showing that [Mn^III^(tmpyp)] is an efficient catalyst for the oxidations of various substrates. High-oxidation-state oxomanganese(IV) and oxomanganese(V) intermediates have been proposed to play significant roles in the process of degradation [34,37,38,39,40,41,42,43,44,45]. H_2_O_2_ as an oxidant, manganese tetra-(*p*-carboxylphenyl) porphyrins (MnP(COOH)_4_), manganese tetra-(*p*-methylpyridium) porphyrins [Mn(TMPyP)], etc., have also been used to catalyze the degradation of azo dyes in the presence of imidazole in aqueous solutions and in nonaqueous solvents [46,47,48]. In a recent study, Meyer et al. reported that an iron porphyrin, *meso*-tetrakis(1-methylpyridinium-4-yl)prophyrinatoiron(III) ([Fe^III^(tmpyp)]), was an efficient catalyst to degrade azo dyes with *meta*-chloroperoxy benzoic acid as an oxidant in an aqueous solution at room temperature [49]. Very recently, our group investigated a ferric porphyrin complex, Fe(III)TCPP, in combination with an oxidant, PhIO, that exhibited a high catalytic efficiency for the degradation of gentian violet [50]. However, very limited iron porphyrins were reported to catalyze the oxidative degradation of triphenylmethane dyes. Herein, we have investigated Fe(III)TCPP for the oxidative degradation of triphenylmethane dyes, including rhodamine B (RhB) and malachite green (MG). The degradation reactions of RhB and MG were monitored with UV-vis absorption spectroscopy via observing the UV-vis spectral changes of these triphenylmethane dyes.

## 2. Results and Discussion

**Figure 1:**
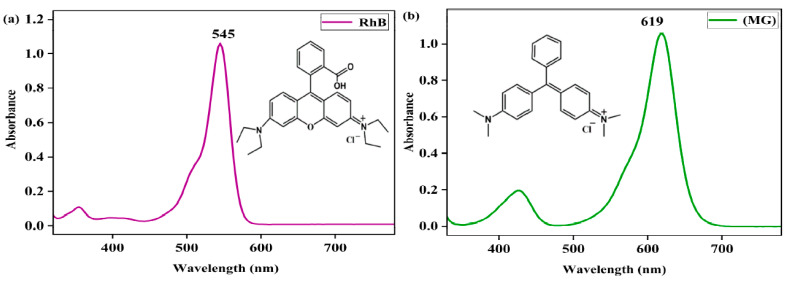
UV-vis spectra of rhodamine B (RhB) (**a**) and malachite green (MG) in MeOH (**b**).

The concentrations of rhodamine B (RhB) and malachite green (MG) in the solution were determined with a UV-vis spectrophotometer with a quartz cuvette (path length = 10 mm) in the wavelength range of 200–800 nm. The UV-vis spectra of 0.05 mM in RhB and MG in MeOH exhibited distinct absorption bands at 545 nm (*λ*_max_) and 619 nm (*λ*_max_), respectively, with absorbances of 1.057 and 1.058 (Figure 1), respectively, and their extinction coefficients (*ε*) were then calculated to be 21,140 M^−1^ cm^−1^ and 21,160 M^−1^ cm^−1^, respectively, using Beer’s Law, *A* = *ε*c, where *A* is the absorbance at 545 nm or 619 nm and c is the concentration of the dye. The concentrations of RhB and MG could be determined from the absorption bands at 545 nm and 619 nm, respectively, following Beer’s Law. The UV-vis spectrum of the Fe(III)TCPP in the MeOH showed a distinct absorption band at 414 nm, which would not have had an influence on the absorption band of gentian violet at 578 nm. Thus, using UV-vis spectroscopy is a suitable way to monitor the concentration changes of gentian violet.

### 2.1. Degradation of Rhodamine B

**Figure 2:**
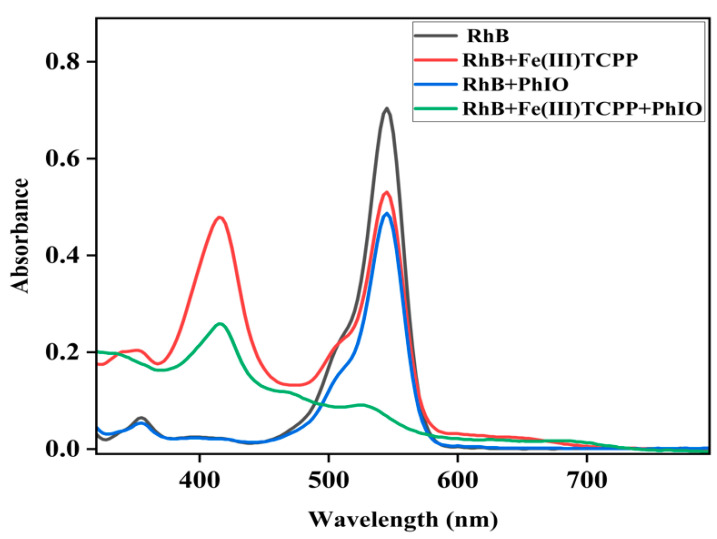
UV-visible absorption spectra of RhB (0.05 mM, black line); the mixture of RhB (0.05 mM) and Fe(III)TCPP (0.0227 mM) after stirring for 30 min (red line); the solution of RhB (0.05 mM) and PhIO (0.4 mM) after stirring for 30 min (blue line) and the solution of RhB (0.05 mM) and PhIO (0.4 mM) in the presence of Fe(III)TCPP (0.0227 mM) after stirring for 30 min (green line).

When the oxidant PhIO was added to the solution of RhB in the presence of Fe(III)TCPP, the color of the solution changed from pink to colorless, which could not be observed without Fe(III)TCPP or PhIO. UV-vis spectroscopy was then used to detect the degradation efficiency of the RhB, as shown in Figure 2, in which little spectral changes can be observed in the absence of Fe(III)TCPP and/or PhIO but a great spectral change can be observed in the presence of both of Fe(III)TCPP and PhIO. Therefore, a system of PhIO and Fe(III)TCPP could be useful to degrade triphenylmethane dye RhB.

**Figure 3:**
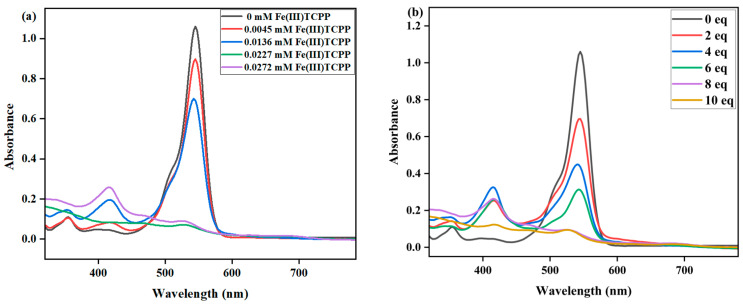
(**a**) UV-vis spectral changes observed in the degradation of RhB at various concentrations of Fe(III)TCPP. Conditions: RhB, 0.05 mM; PhIO, 0.4 mM; Fe(III)TCPP, 0.00454 mM, 0.01362 mM, 0.0227 mM and 0.02772 mM; solvent, MeOH; 303 K; reaction time, 30 min. (**b**) UV-vis spectral changes observed in the degradation of RhB at various concentrations of PhIO. Conditions: RhB, 0.05 mM; Fe(III)TCPP, 0.0227 mM; PhIO, 2 eq, 4 eq, 6 eq, 8 eq and 10 eq; solvent, MeOH; 303 K; reaction time, 30 min.

The effect of Fe(III)TCPP concentrations on the degradation of RhB was first studied with fixed concentrations of PhIO and RhB in the Fe(III)TCPP/PhIO system. A typical set of Fe(III)TCPP concentration-dependent spectral absorptions recorded for the Fe(III)TCPP-catalyzed degradation of RhB in MeOH is shown in Figure 3a. When the concentration of Fe(III)TCPP increased, the absorbance band at 545 nm for the RhB decreased, and it gave more than a 90% degradation efficiency when 0.0227 mM of Fe(III)TCPP was added (Figure 3a, Table 1). There was no big change when more than 0.0227 mM of Fe(III)TCPP was added. Spectral measurements confirmed that the presence of both Fe(III)TCPP and PhIO is essential for the degradation of RhB and a high concentration of Fe(III)TCPP is beneficial to improving degradation efficiency.

**Figure 4:**
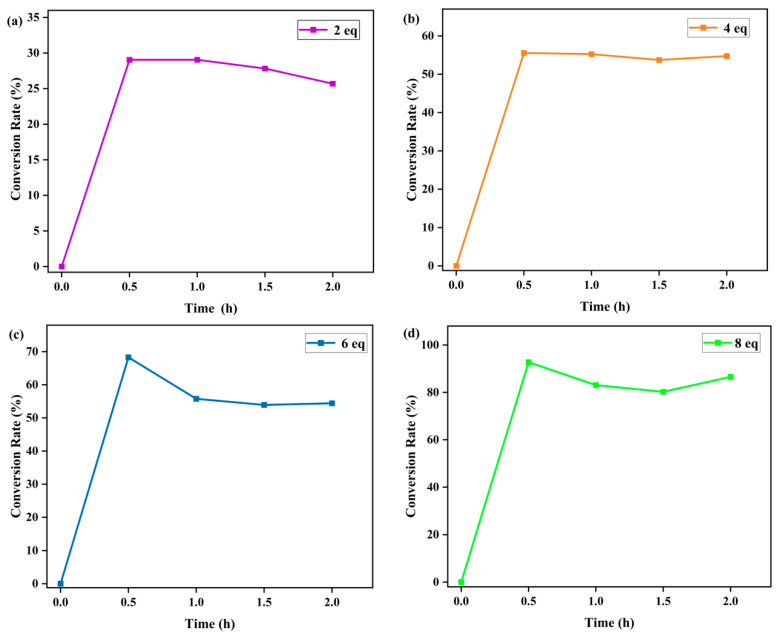
Effect of reaction time on the Fe(III)TCPP-catalyzed degradation of gentian violet with 2 eq (**a**), 4 eq (**b**), 6 eq (**c**) and 8 eq (**d**) of PhIO. Conditions: RhB, 0.05 mM; Fe(III)TCPP, 0.0227 mM; solvent, MeOH; 303 K.

The effects of PhIO concentration and reaction time on the degradation of RhB were then studied at fixed concentrations of Fe(III)TCPP and RhB in a Fe(III)TCPP/PhIO system (Figure 3b and Figure 4). A typical set of PhIO-concentration-dependent spectral absorptions recorded for the Fe(III)TCPP-catalyzed degradation of RhB in MeOH at 303 K was observed, as shown in Figure 3b and Table 1. The absorbance band at 545 nm for the RhB decreased when the concentration of PhIO was increased. When 2 eq of PhIO was added, the degradation efficiency of the RhB was the lowest and was kept basically unchanged after 30 min. When 4 eq and 6 eq of PhIO were used, the degradation efficiencies increased to 56% and 68%, respectively, within 30 min. When 8 eq of oxidant was added, the degradation efficiency of the RhB increased to 93% within 30 min, showing a much higher degradation efficiency than that of photocatalytic oxidation using the titanium molybdate nanoparticles as a photocatalyst under solar irradiation, in which only around a 42% degradation efficiency was obtained within 30 min [51], and a comparable degradation efficiency to the photocatalytic oxidation was shown when crosslinked gelatin/CuS/PVA nanocomposites were used as photocatalysts under solar irradiation, in which the catalysts retained their efficiency and the rate of the degradation process was still above 80% after each catalyst was used five times sequentially [3]. No more changes were observed at 545 nm when more than 8 eq of PhIO was added. All degradation reactions were finished within 30 min. The degradation product of the RhB produced by the Fe(III)TCPP/PhIO system was detected with GC-MS. However, only iodobenzene (PhI) with a molecular weight of 204 was shown in the GC-MS, coming from the reduction of the PhIO. No product of the RhB was observed, which may have been due to the formation of high-boiling-point products, resulting in no detection with GC-MS.

### 2.2. Degradation of Malachite Green (MG)

**Figure 5:**
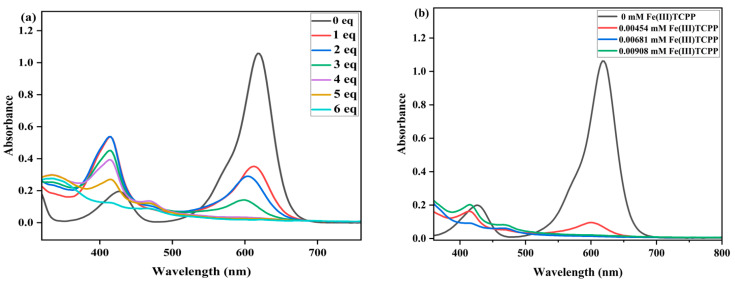
(**a**) UV-vis spectral changes observed in the degradation of MG at various concentrations of PhIO. Conditions: MG, 0.05 mM; Fe(III)TCPP, 0.01816 mM; PhIO, 1 eq, 2 eq, 3 eq, 4 eq, 5 eq and 6 eq; solvent, MeOH; 303 K; reaction time, 30 min. (**b**) UV-vis spectral changes observed in the degradation of MG with various concentrations of catalyst. Conditions: MG, 0.05 mM; PhIO, 5 eq; Fe(III)TCPP, 0.00454 mM, 0.00681 mM and 0.00908 mM; solvent, MeOH; 303 K; reaction time, 30 min.

Another triphenylmethane dye, malachite green (MG), was selected to detect the catalytic degradation efficiency of the Fe(III)TCPP/PhIO system. The green color of the solution transitioned to colorlessness when the PhIO was added to the solution of MG in the presence of Fe(III)TCPP, indicating that the system of PhIO and Fe(III)TCPP can be a useful platform for degrading MG. The effect of PhIO concentration on the degradation of MG was first studied with fixed concentrations of Fe(III)TCPP and MG in the Fe(III)TCPP/PhIO system. A typical set of PhIO-concentration-dependent spectral absorptions recorded for the Fe(III)TCPP-catalyzed degradation of MG is presented in Figure 5a. When the concentration of PhIO increased, the absorbance band at 619 nm for the MG decreased, and this gave more than a 95% degradation efficiency when 5 eq of PhIO was added (Figure 5a, Table 2), showing a remarkable degradation of the MG. This was kept unchanged when more than 5 eq of PhIO was added.

The effect of Fe(III)TCPP concentration was then explored at fixed concentrations of PhIO and MG in a Fe(III)TCPP/PhIO system (Figure 5b). A typical set of catalyst-concentration-dependent spectral absorptions recorded for the Fe(III)TCPP-catalyzed degradation of the MG in MeOH at 303 K was observed, as shown in Figure 5b and Table 2. The absorbance band at 916 nm for the MG decreased dramatically when the concentration of Fe(III)TCPP was increased. When 0.00454 mM of Fe(III)TCPP was used, the degradation efficiency of the MG reached up to 94% in 30 min, and it was up to 97% when 0.00681 mM of the catalyst was used.

**Figure 6:**
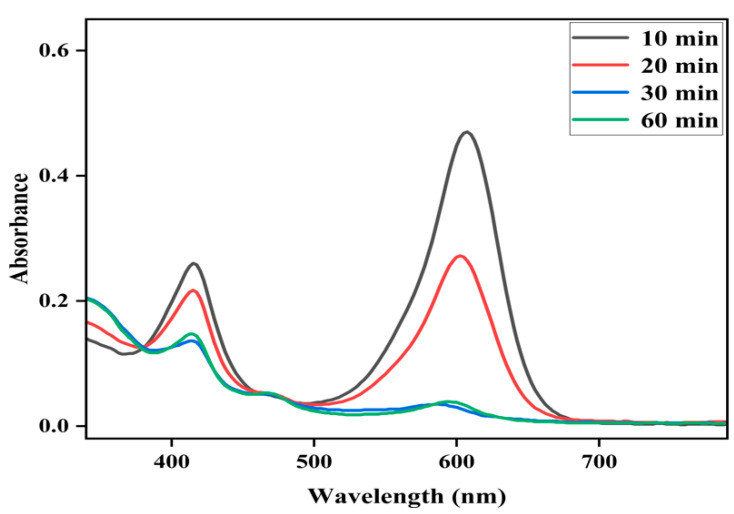
Effect of reaction time on the Fe(III)TCPP-catalyzed degradation of MG with PhIO. Conditions: MG, 0.05 mM; Fe(III)TCPP, 0.00681 mM; PhIO, 5 eq; solvent, MeOH; 303 K.

**Figure 7:**
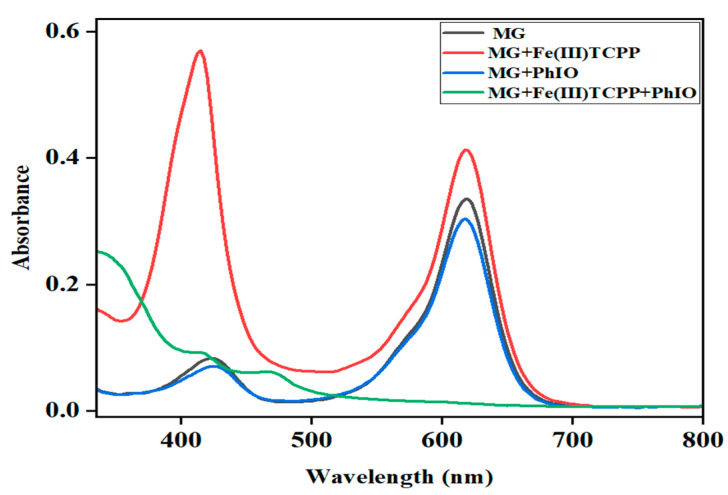
UV-visible absorption spectra of MG (0.05 mM, black line); the mixture of MG (0.05 mM) and Fe(III)TCPP (0.00681 mM) after stirring for 30 min (red line); the solution of MG (0.05 mM) and PhIO (5 eq) after stirring for 30 min (blue line) and the solution of MG (0.05 mM) and PhIO (5 eq) in the presence of Fe(III)TCPP (0.00681 mM) after stirring for 30 min (green line).

The effect of reaction time on the degradation of MG was further examined at fixed concentrations of Fe(III)TCPP, PhIO and MG, and the results are presented in Figure 6. As shown in Figure 6, when reacting for 10 min, the degradation efficiency of the MG was 61%. When reacting for 30 min, the absorbance band at 916 nm for the MG decreased dramatically and the degradation efficiency was up to more than 95%, showing a rapid kinetic degradation efficiency of the MG. There were no big changes kept when the reaction time was more than 30 min. The control reactions were carried out to highlight the importance of the Fe(III)TCPP/PhIO system when reacting for 30 min (Figure 7). Little spectral changes were observed in the absence of Fe(III)TCPP and/or PhIO, while great spectral changes were observed in the presence of both of Fe(III)TCPP and PhIO, in which more than 95% of the degradation efficiency was obtained, showing a comparable degradation efficiency to the photocatalytic oxidation when rGO-Fe_3_O_4_/TiO_2_ was used as a photocatalyst under visible light for MG degradation [14]. The degradation products of the MG in the Fe(III)TCPP/PhIO system were also detected with GC-MS, and the results were the same as for the RhB.

**Figure 8:**
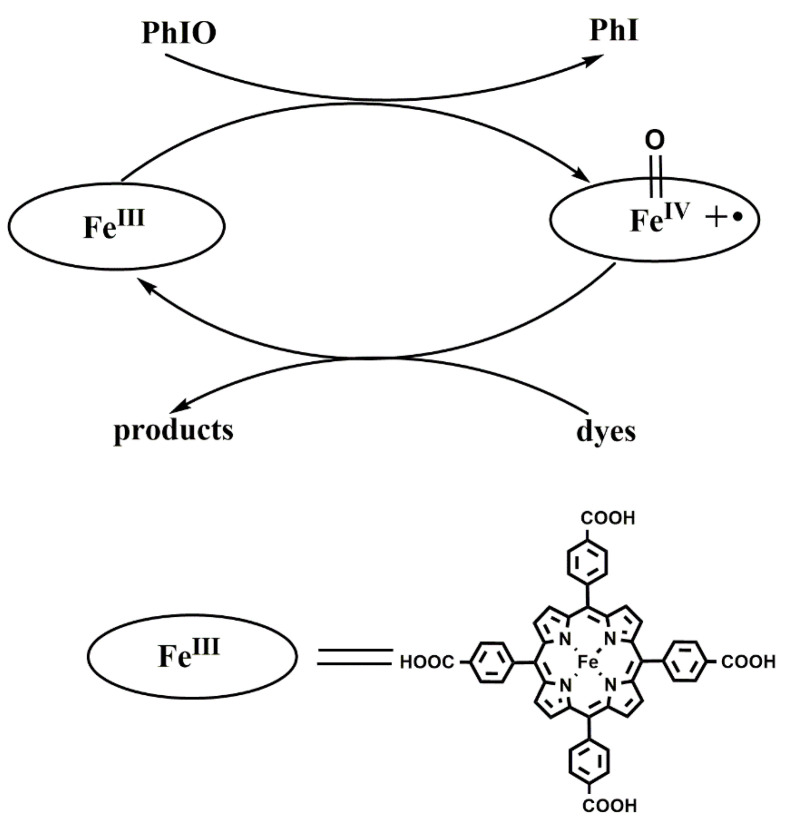
Proposed mechanism for the catalytic degradation of dyes.

The above experimental results have indicated that Fe(III)TCPP is a useful catalyst, in combination with the oxidation of PhIO, to degrade triphenylmethane dyes. It is generally believed that iron(IV)-oxo porphyrin π-cation radical species [Fe^IV^(O)(porph^+•^)] are active species and play important parts in the process of the degradation of dyes. Meyer reported the oxidative degradation of azo dyes with [Fe^III^(tmpyp)], with *meta*-chloroperoxy benzoic acid as an oxidant, at room temperature and concluded that [Fe^IV^(O)(tmpyp^+•^)] could oxidize and decompose the naphthalene cores of azo dyes [49]. Kong proposed that an iron(IV)-oxo porphyrin π-cation radical produced by heme-catalyzed O-O bond heterolysis in H_2_O_2_ may play a major part in dye degradation [52]. Therefore, based on the previously proposed mechanism [49,52] and the above results obtained from the various experiments with the Fe(III)TCPP/PhIO system, we have hypothesized that the reaction between Fe(III)TCPP and PhIO gives transient, intermediate [Fe^IV^(O)(TCPP^+•^)] (Figure 8). [Fe^IV^(O)(TCPP^+•^)] rapidly interacts with triphenylmethane dyes, resulting in the degradation of these dyes and the regeneration of the catalyst Fe(III)TCPP.

## 3. Materials and Methods

### 3.1. Materials and Instruments

All reagents and solvents were commercially purchased and used without further purification. Rhodamine B (RhB) (Tianjin Damao Chemical Reagent Factory, Tianjin, China), malachite green (MG) (Shanghai Maclean Biochemical Technology Co., Ltd., Shanhai, China) and 5,10,15,20-tetra(4-carboxyphenyl)porphyrinato iron(III) chloride (Fe(III)TCPP; Shanghai bide Pharmaceutical Technology Co., Ltd., Shanghai, China) were used as provided. (Diacetoxyiodo)benzene (PhI(OAc)_2_) was provided by Shanghai McLean Biochemical Technology Co., Ltd., Shanghai, China. The water used in all experiments was distilled. Iodosylbenzene (PhIO) was prepared based on a previously reported method in the literature [53]. UV-vis absorption spectra were recorded on a Hitachi U-3010 spectrometer.

### 3.2. Degradation of Dyes

The stock solutions of the dyes, with concentrations of 10 mM, and the Fe(III)TCPP, with a concentration of 0.227 mM, were prepared, respectively, in MeOH and were kept for further use. It should be noted that the stock solution of the PhIO (10 mM) was reconfigured with each use.

#### 3.2.1. Catalytic Oxidative Degradation of Rhodamine B

The effects of catalyst concentration were as follows: 0.1 mL (0.00454 mM), 0.3 mL (0.01362 mM), 0.5 mL (0.0227 mM) and 0.6 mL (0.02724 mM) of the stock solution of Fe(III)TCPP, respectively; 0.025 mL (0.05 mM) of the stock solution of RhB; and 0.2 mL (0.4 mM) of the stock solution of PhIO were put in 5 mL of MeOH. The reaction solution was stirred in a water bath at a constant temperature of 30 °C for 30 min, and the concentration of RhB in the resulting solution was determined with a UV–vis spectrophotometer.

The effect of PhIO concentration was as follows: 0.025 mL (0.05 mM) of the stock solution of RhB and 0.5 mL (0.0227 mM) of the stock solution of Fe(III)TCPP were put in 5 mL of MeOH. Then, 2 eq, 4 eq, 6 eq, 8 eq and 10 eq of PhIO (relative to the RhB; same as below) were added into the solution, respectively. The reaction solution was stirred at a constant temperature of 30 °C for 30 min, and the concentration of RhB in the resulting solution was determined with a UV–vis spectrophotometer.

For the control experiments, 0.025 mL (0.05 mM) of the stock solution of RhB and 0.5 mL (0.0227 mM) of the stock solution of Fe(III)TCPP or 8 eq of PhIO were put in 5 mL of MeOH. The reaction solution was stirred at a constant temperature of 30 °C for 30 min, and the concentration of RhB in the resulting solution was determined with a UV–vis spectrophotometer.

#### 3.2.2. Catalytic Oxidative Degradation of Malachite Green

The effects of catalyst concentration were as follows: 0.2 mL (0.00454 mM, 9%), 0.3 mL (0.00681 mM) and 0.4 mL (0.00908 mM) of the stock solution of Fe(III)TCPP, respectively; 0.05 mL (0.05 mM) of the stock solution of MG; and 0.25 mL (0.25 mM) of the stock solution of PhIO were put in 10 mL of MeOH. The reaction solution was stirred in a water bath at a constant temperature of 30 °C for 30 min, and the concentration of MG in the resulting solution was determined with a UV–vis spectrophotometer.

The effect of PhIO concentration was as follows: 0.025 mL (0.05 mM) of the stock solution of MG and 0.5 mL (0.0227 mM) of the stock solution of Fe(III)TCPP were put in 5 mL of MeOH. Then, 1 eq, 2 eq, 3 eq, 4 eq, 5 eq and 6 eq of PhIO were added into the solution, respectively. The reaction solution was stirred at a constant temperature of 30 °C for 30 min, and the concentration of MG in the resulting solution was determined with a UV–vis spectrophotometer.

Control reactions: 0.025 mL (0.05 mM) of the stock solution of MG and 0.15 mL (0.00681 mM) of the stock solution of Fe(III)TCPP or 5 eq of PhIO were put in 5 mL of MeOH. The reaction solution was stirred at a constant temperature of 30 °C for 30 min, and the concentration of MG in the resulting solution was determined with a UV–vis spectrophotometer.

## 4. Conclusions

Various methods have been developed and widely applied for the degradation of dyes. However, these traditional methods have certain limitations. As the representative model of cytochrome P450, metalloporphyrin has incomparable catalytic specificity and efficiency and can be used as a catalyst to overcome the shortcomings of dye degradation methods, as mentioned above. In this work, the potential of the iron porphyrin complex Fe(III)TCPP to degrade triphenylmethane dyes was investigated; Fe(III)TCPP was combined with the oxidant PhIO to degrade RhB and MG. The concentrations of RhB and MG were monitored with UV-vis absorption spectroscopy. The Fe(III)TCPP/PhIO system had a high catalytic degradation efficiency, and the degradation efficiency reached more than 95% in 30 min at 303 K. Thus, an iron porphyrin as the cytochrome P450 model can play an important part in the degradation of triphenylmethane dyes, providing an efficient approach for the treatment of wastewater from dyeing.

## Figures and Tables

**Table 1:** Fe(III)TCPP-catalyzed degradation ^1^ of RhB with PhIO.

[Fe(III)TCPP]	PhIO (eq)	Degradation Efficiency (%)
0.0227	-	25
-	8	31
0.0136	8	35
0.0227	8	93
0.0272	8	93
0.0227	2	29
0.0227	4	56
0.0227	6	68
0.0227	8	93
0.0227	10	93

^1^ Conditions: RhB, 0.05 mM; Fe(III)TCPP, 0.0227 mM; PhIO, 0, 2, 4, 6, 8 and 10 equiv; solvent, MeOH; 303 K; reaction time, 30 min.

**Table 2:** Fe(III)TCPP-catalyzed degradation^1^ of MG with PhIO.

Fe(III)TCPP	PhIO (eq)	Degradation Efficiency (%)
0.00681	-	18
-	5	30
0.00454	5	93
0.00681	5	98
0.00908	5	98
0.00681	1	57
0.00681	2	70
0.00681	3	88
0.00681	4	96
0.00681	5	97
0.00681	6	98

^1^ Conditions: MG, 0.05 mM; Fe(III)TCPP, 0.00681 mM; PhIO, 0, 1, 2, 3, 4, 5 and 6 eq; solvent, MeOH; 303 K; reaction time, 30 min.

## Data Availability

Not applicable.
